# The complete chloroplast genome of *Ageratum conyzoides* (Asteraceae)

**DOI:** 10.1080/23802359.2019.1673241

**Published:** 2019-10-04

**Authors:** Zi-Peng Qiao, Zhi-Xiang Chen, Qi-Zhi Wang

**Affiliations:** College of Chemical Engineering, Huaqiao University, Xiamen, PR China

**Keywords:** *Ageratum conyzoides* L., complete chloroplast genome, phylogenetic analysis

## Abstract

*Ageratum conyzoides* L. is an important Chinese medicinal plant. In this study, we reported the complete chloroplast genome of *A*. *conyzoides*. The chloroplast genome sequence is 151,309 bp in length and consisted of a large single copy (LSC) region (83,884 bp), a small single copy (SSC) region (17,771 bp), and two inverted repeats (IRs) (24,827 bp). It was composed of 126 genes and they were 81 protein-coding genes, 30 tRNA genes, 8 rRNA genes, and 7 pseudogene. Phylogenetic analysis with reported chloroplast genomes can not only show that *A. conyzoides* has a close genetic relationship with *Centaurea diffusa* and *Carthamus tinctorius*, but also provide new evidence for the identification of *Praxelis clematidea* and *A. conyzoides*.

*Ageratum conyzoides* L., is an annual herb with a long history of traditional medicinal uses in many countries in the world, especially in the tropical and subtropical regions (Okunade 2002). The extracts and metabolites of *A. conyzoides* have been used as a bacteriocide, antidysentric, anti-diabetic, insecticide, and herbicide (Borthakur and Baruah 1987; Xuan et al. 2004; Nogueira et al. 2010). However, due to its purple floret, *A*. *conyzoides* is sometimes difficult to distinguish from other purple floret plant, such as *Praxelis clematidea*. As such, obtaining the complete chloroplast sequence will enable identification by molecular markers.

Fresh leaves of *A. conyzoides* collected from Huaqiao University (31°15N, 109°56E), Fujian Province, China. Voucher specimens were deposited in Huaqiao University Herbarium (18014011). Total genomic DNA was extracted by PlantGenomic DNA Kit (Sangon Biotech, Shanghai, China). Paired-end reads were sequenced by using Illumina Hiseq Platform (Illumina, San Diego, CA). Approximately 10 Gb of paired-end (150 bp) sequence data were randomly extracted from the total sequencing output and used as input for NOVOPlasty (Dierckxsens et al. 2017) to assemble the plastid genome. The plastid genome of *P*. *clematidea* (GenBank accession number: NC_023833.1) was used as the seed sequence. The assembled sequence was annotated using Geneious version 11.0.4 (Kearse et al. 2012) by comparing it with the complete chloroplast genomes of *P*. *clematidea*. At last, the annotated chloroplast genome sequence was submitted to GenBank with the accession number MK905238.

The *A. conyzoides* chloroplast genome was 151,309 bp in length with an overall GC content of 37.4%. The circle genome was comprised a large single copy (LSC) region (83,884 bp), a small single copy (SSC) region (17,771 bp), and two inverted repeats (IRs) (24,827 bp). The complete chloroplast genome contains 126 genes, including 81 protein-coding genes, 30 tRNA genes, 8 rRNA, genes, and 7 pseudogene.

A neighbour-joining (NJ) tree was performed with EGA7.0 (Kumar et al. 2016) based on 13 complete chloroplast genome sequences of Asteraceae and *Platycodon grandiflorus* (Campanulaceae) (NC 035624) as an outgroup using 1000 bootstrap replicates (Figure 1). The tree showed a close relationship between *Centaurea diffusa* and *Carthamus tinctorius*. This complete cp genome can not only be further used for population genomic studies, phylogenetic analyses and genetic engineering studies of Asteraceae, but also provide new evidence for the identification of *P. clematidea* and *A. conyzoides*.

**Figure 1.**
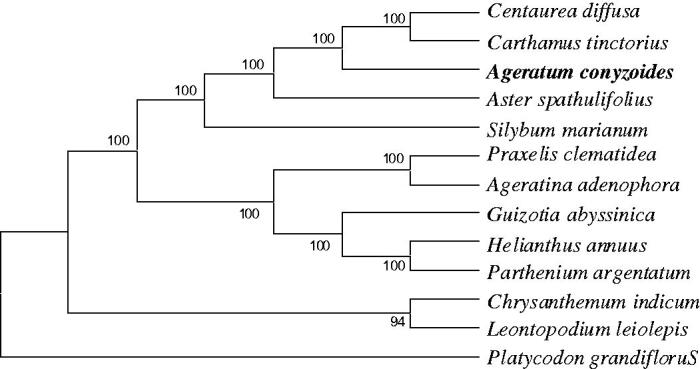
Neighbour-joining (NJ) phylogenetic tree based on 14 complete chloroplast genomes. Accession numbers: *Ageratum conyzoides* (MK905238); *Aster spathulifolius* (NC_027434); *Centaurea diffusa* (NC_024286); *Carthamus tinctorius* (NC_030783); *Silybum marianum* (NC_028027); *Praxelis clematidea* (KF922320); *Ageratina adenophora* (NC_015621); *Guizotia abyssinica* (NC_010601); *Helianthus annuus* (NC_007977); *Parthenium argentatum* (NC_013553); *Chrysanthemum indicum* (JN867589); *Leontopodium leiolepis* (NC_027835); and *Platycodon grandiflorus* (NC_035624).
